# Supplementary material for: Decoding the News Media Diet of Disinformation Spreaders
Source: Entropy (Basel). 2024 Mar 19;26(3):270. doi: 10.3390/e26030270 (PMC10969463; doi:10.3390/e26030270)
Supplement: Supplementary file 1 [file entropy-26-00270-s001.zip › entropy-2877431-supplementary.pdf]

Supplementary Information: Decoding the News Media Diet of Disinformation Spreaders

Anna Bertani, Valeria Mazzeo, Riccardo Gallotti

Levels of High-Risk users

We therefore choose to distinguish users between those only sharing reliable (Mainstream Media, Science) and Low Risk news (Satire, Other, Political, Click-bait), and those sharing high risk content (Fake/Hoax, Conspiracy/Junkscience), and then differentiating further based on the increasing engagement with this type of high risk content. For this reason, we decided to classify the users into four categories, highlighting their increasing interaction with high risk sources. For the purpose of comparison, we decided to perform the same analysis, taking into account other levels of interactions with high risk news media sources in order to understand if the same pattern is recognizable in such a scenario. Our initial result is confirmed with the ones found when considering several levels of high risk sources as shown in Figure 1.

Verified accounts

In order to differentiate between single users accounts and accounts managed by multiple users, we decided to perform the same analysis by comparing users classified as verified and those classified as unverified. In this context, Verified users are those having the blue verified badge, a blue check mark, that defines those accounts that are of public interest because they are considered authentic, notable and active on Twitter. The verification process of users is given by the blue check mark that can be found next to the username, while unverified accounts do not have this distinctive signal. However, this definition pertains to the period before Musk's takeover, during which accounts were required to undergo request verification. Starting from April 1st, 2023, there has been a change in the rule. For users to acquire the verification badge now, they are required to subscribe to Twitter Blue. Regarding this research, we adhere to the initial definition since the new rule was not in effect during the considered time frame. However, we found that the verified accounts are almost 2% of our dataset, sharing mainly reliable information, few accounts engage with high-risk news media content. Also in this case, we observe that both verified users and unverified users show a similar pattern especially when comparing the random

entropy and the actual entropy, demonstrating again that users sharing unreliable content have a more varied diet but more regular compared with users sharing only reliable and low-risk content.

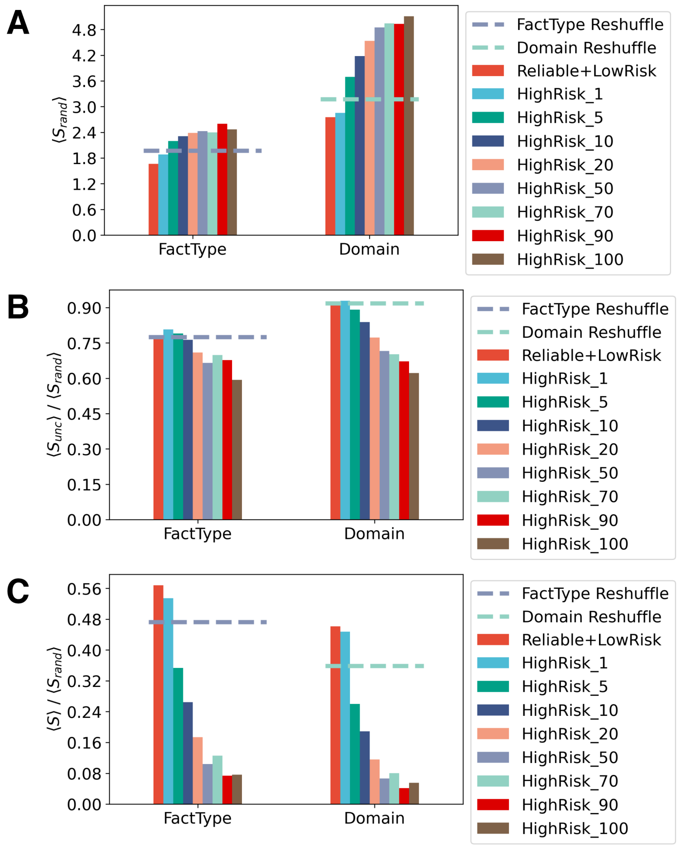

Supplementary Figure S1: **News Media Diet for different type of users (9 categories of users).** **(A)** Random Entropy S_{rand} calculated for different type of accounts: users posting reliable or low-risk content and users posting different levels of high risk (conspiracy/junk science and/or fake/hoax) content.**(B)** Shannon Entropy S_{unc} calculated on the domain and the type of news shared by different type of users accounts: users posting reliable or low-risk content and users posting different levels of high risk (conspiracy/junk science and/or fake/hoax) content. **(C)** The actual entropy S calculated on the do-main and news media categories shared by different type of users: those posting reliable or low-risk content and those posting different levels of high risk (con-spiracy/junk science and/or fake/hoax) content.

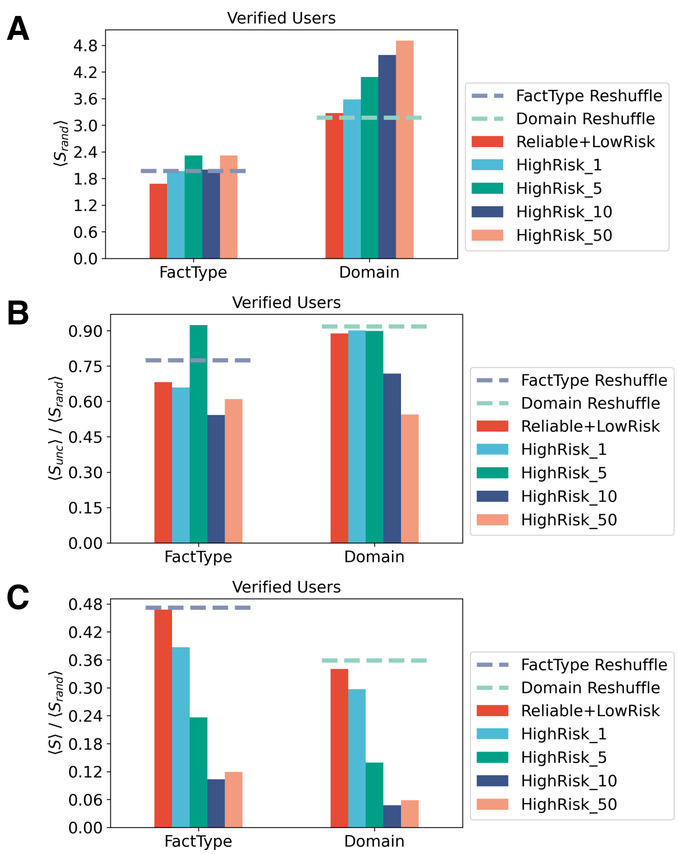

Supplementary Figure S2: **News Media Diet for verified users. (A)** Random Entropy S_{rand} calculated for different type of accounts: users posting reliable or low-risk content and users posting different levels of high risk (con-spiracy/junk science and/or fake/hoax) content. **(B)** Shannon Entropy S_{unc} calculated on the domain and the type of news shared by different type of users accounts: users posting reliable or low-risk content and users posting different levels of high risk (conspiracy/junk science and/or fake/ hoax) content. **(C)** The actual entropy S calculated on the domain and news media categories shared by different type of users: those posting reliable or low-risk content and those posting different levels of high risk (conspiracy/ junk science and/or fake/hoax) content.

1 Cross-countries Analysis

In our analyses, we consider an aggregated sample of 25 countries, having at least 500 tweets on average per day, highlighting a sort of collective pattern across countries. For the purpose of comparison, we decided to take into con-

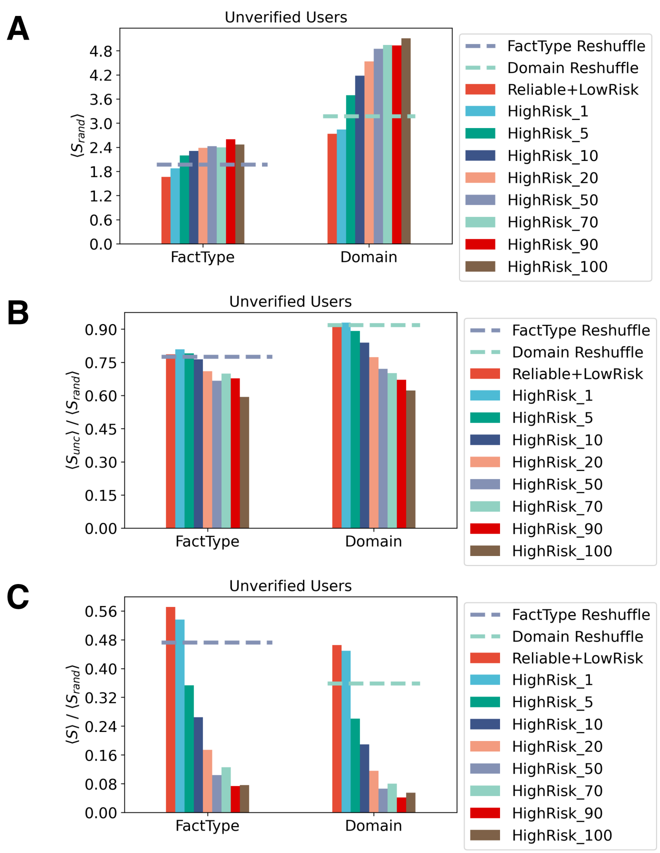

Supplementary Figure S3: **News Media Diet for users classified as unver-ified.** **(A)** Random Entropy S_{rand} calculated for different type of accounts: users posting reliable or low-risk content and users posting different levels of high risk (conspiracy/junk science and/or fake/hoax) content. **(B)** Shannon Entropy S_{unc} calculated on the domain and the type of news shared by differ-ent type of users accounts: users posting reliable or low-risk content and users posting different levels of high risk (conspiracy/junk science and/or fake/hoax) content. **(C)** The actual entropy S calculated on the domain and news media categories shared by different type of users: those posting reliable or low-risk content and those posting different levels of high risk (conspiracy/junk science and/or fake/hoax) content.

sideration two different countries to explore the differences or similarities among them with respect to the results found in our analysis. Drawing inspiration from World Press Freedom[1] Index, an index measuring the freedom of the press in 180 countries world- wide, we compared Colombia with a lower value (45,23)

[1] https://rsf.org/en/index

against the Netherlands having a much bigger value (87). We found that both countries show a similar pattern, taking into consideration also the values observed when aggregating all the countries considered in the study. Indeed, the news media environment (inter-relations among different news media categories) is characterized by having a strong relationship with mainstream media journals and political news, when we look at the value not normalized. On the contrary, the relations between fake and conspiracy news is much stronger when comparing our results with the null model. Interestingly, we found that this relation is much stronger for Colombia rather than the Netherlands. Indeed, the Netherlands show a much stronger relation between Fake and Political News when analyzing our results normalized. We investigated also the news media diet by comparing the three different measures of entropy (Random, Uncorrelated and the actual entropy), demonstrating that the results are consistent with the ones found in the aggregated analysis and among the two different countries.

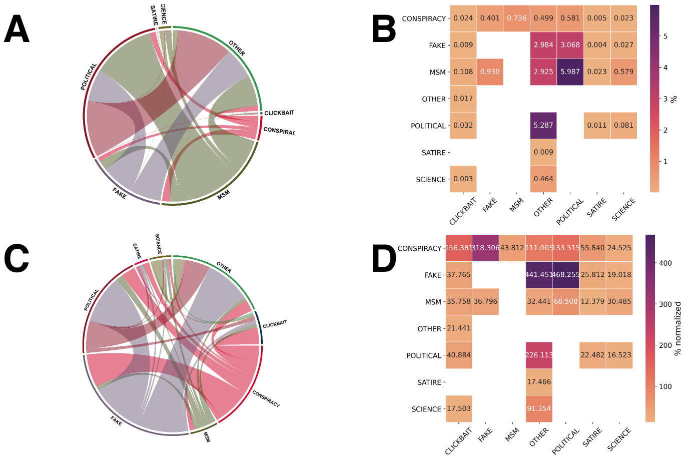

Supplementary Figure S4: **The News Media Environment (The Nether-lands).** **(A)** Weighted networks of the interactions among different news me-dia types (also known as intra-relations) for 25 countries in 2020. **(B)** Heatmap showing the corresponding value of the inter-relations among the eight cate-gories. **(C)** Weighted networks normalized comparing with a null model ac-counting for the proportion of the number of news belonging to each category. The chances of sharing conspiracy and fake news by the same users is much higher than the strong relation observed between mainstream media and politi-cal news (Panel A). **(D)** Heatmap showing the corresponding normalized value of the inter-relations among the eight categories.

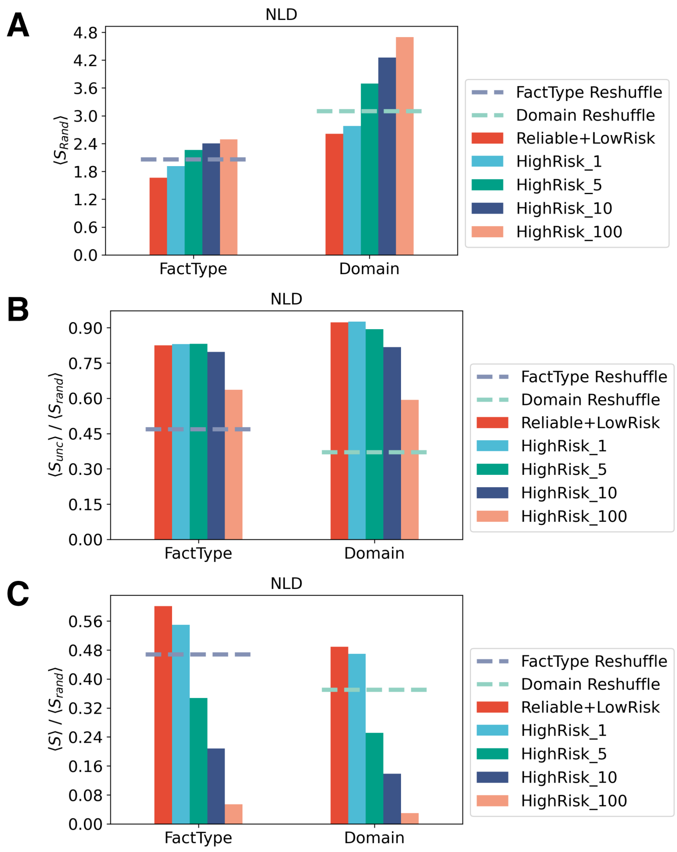

Supplementary Figure S5: **News Media Diet for different type of users (The Netherlands).** **(A)** Random Entropy S_{rand} calculated for different type of accounts: users posting reliable or low-risk content and users posting different levels of high risk (conspiracy/junk science and/or fake/hoax) content. **(B)** Shannon Entropy S_{unc} calculated on the domain and the type of news shared by different type of users accounts: users posting reliable or low-risk content and users posting different levels of high risk (conspiracy/junk science and/or fake/hoax) content. **(C)** The actual entropy S calculated on the domain and news media categories shared by different type of users: those posting reliable or low-risk content and those posting different levels of high risk (conspiracy/junk science and/or fake/hoax) content.

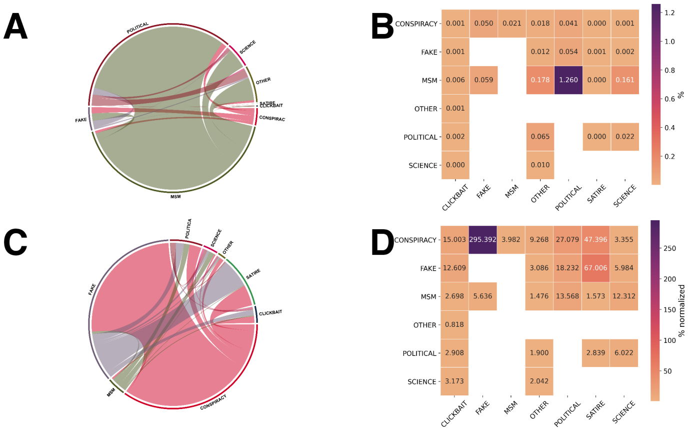

Supplementary Figure S6: **The News Media Environment (Colombia).(A)** Weighted networks of the interactions among different news media types (also known as intra-relations) for 25 countries in 2020. **(B)** Heatmap showing the corresponding value of the inter-relations among the eight categories. **(C)** Weighted networks normalized comparing with a null model accounting for the proportion of the number of news belonging to each category. The chances of sharing conspiracy and fake news by the same users is much higher than the strong relation observed between mainstream media and political news (Panel A). **(D)** Heatmap showing the corresponding normalized value of the inter-relations among the eight categories.

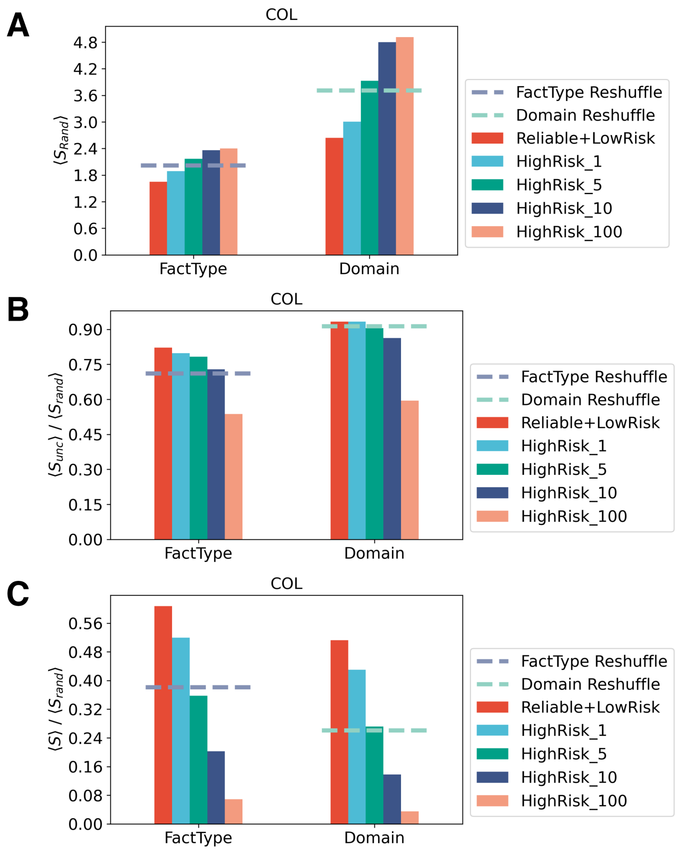

Supplementary Figure S7: **News Media Diet for different type of users (Colombia).** **(A)** Random Entropy S_{rand} calculated for different type of accounts: users posting reliable or low-risk content and users posting different levels of high risk (conspiracy/junk science and/or fake/hoax) content. **(B)** Shannon Entropy S_{unc} calculated on the domain and the type of news shared by different type of users accounts: users posting reliable or low-risk content and users posting different levels of high risk (conspiracy/junk science and/or fake/hoax) content. **(C)** The actual entropy S calculated on the domain and news media categories shared by different type of users: those posting reliable or low-risk content and those posting different levels of high risk (conspiracy/junk science and/or fake/hoax) content.
